# New Insights into the Basic and Translational Aspects of AMPK Signaling

**DOI:** 10.3390/cells12020206

**Published:** 2023-01-04

**Authors:** Yury Ladilov, Muhammad Aslam

**Affiliations:** 1 Department of Cardiovascular Surgery, Heart Center Brandenburg, University Hospital Brandenburg, Brandenburg Medical School Theodor Fontane, Ladeburger Str. 17, 16321 Bernau, Germany; 2 Experimental Cardiology, Department of Internal Medicine I, Justus Liebig University, Aulweg 129, 35392 Giessen, Germany; 3 Department of Cardiology, Kerckhoff Clinic GmbH, 61231 Bad Nauheim, Germany; 4 DZHK (German Centre for Cardiovascular Research), Partner Site Rhein-Main, 61231 Bad Nauheim, Germany

5′-adenosine monophosphate (AMP)-activated protein kinase (AMPK) is an enzyme regulating numerous cellular processes involved in cell survival as well as health- and lifespan. AMPK has emerged not only as a key cellular energy sensor but also as an important integrator of signals that manages cellular energy balance. Due to this property and being ubiquitously expressed in all mammalian cell types, AMPK has attracted interest in virtually all areas of biomedical research. In this Special Issue of *Cells*, several up-to-date reviews and original studies have provided new insights into the basic and translational aspects of AMPK signaling.

## 1. Basic Aspects

Visnjic et al. [1] considered an important methodological approach in AMPK research by giving an overview of the widely used AMPK activator 5-amino-4-imidazolecarboxamide ribonucleoside (AICAr). In particular, the authors described its AMPK-dependent and AMPK-independent effects covering basic aspects for both, such as metabolism, nucleotide synthesis, cell cycle, and diseases, e.g., ischemia, diabetes, and cancer.

In the other review paper, Aslam and Ladilov [2] described the pathways that link cAMP and AMPK. Since many of the physiological stresses lead to cellular cAMP elevation and are associated with increased energy consumption, it is not surprising that the activation of cAMP signaling may promote AMPK activity. The authors described several mechanisms leading to the activation of the AMP-AMPK axis and its beneficial role in mitochondrial homeostasis, lipid metabolism, and inflammation regulation, as well as in diseases such as ischemia and diabetes. An important translational message of the review is that physical activity leading to the elevation of cellular cAMP levels may be a “drug-free” approach to promoting the cAMP-AMPK axis.

Obesity has been categorized by the American Medical Association as a chronic progressive metabolic disorder and is a growing public health concern worldwide. In the paper by van der Vaart et al. [3], an important approach for fighting obesity was reviewed. The authors described the basic mechanisms of brown adipose tissue (BAT) activation and investigated how AMPK can be used as a target for BAT activation. In particular, several AMPK-mediated signaling pathways involved in BAT activation were presented in the review. These pathways control three main processes involved in BAT activation: the development of brown adipocytes, support for mitochondrial health, and increased thermogenesis.

Finally, an original study by Li et al. [4] provided new data describing the role of AMPK in the effects of C1q tumor necrosis factor-alpha-related proteins (CTRPs) on glucose and fatty acid metabolism in adult rat cardiomyocytes and H9C2 cardiomyoblasts. Among several CTRPs leading to the phosphorylation of AMPK, only CTRP2, 7, 9, and 13 induced GLUT1 and GLUT4 translocation and glucose uptake, as well as the upregulation of enzymes involved in glucose or fatty acid metabolism. Since the knockdown of the adiponectin receptor 1 abolished CTRP7- and CTRP9-mediated phosphorylation of AMPK and ACC, the authors suggest a major role of this receptor in promoting AMPK activation via CTRPs.

## 2. Translational Aspects

In a sophisticated study, Olivier et al. [5] investigated the intestinal epithelial protective role of AMPK in colitis. A deficiency of AMPK in the intestinal epithelial cells delayed epithelial repair in a mouse model of colitis. On the other hand, metformin, an anti-diabetic drug activating AMPK, accelerated intestinal repair [5,6]. Interestingly, diabetic patients using metformin to control their blood glucose level are at a lower risk of inflammatory bowel disease compared to diabetic patients using other anti-diabetic drugs [7]. To explore this phenomenon further, a number of clinical trials have been registered to use metformin as an add-on/adjuvant therapy in ulcerative colitis [8,9,10].

Non-alcoholic fatty liver disease (NAFLD) is emerging as a leading cause of chronic liver illness affecting > 30% of the European population, and even higher rates can be seen in the US population [11,12]. The hallmark finding that AMPK regulates fatty acid metabolism by inactivating acetyl CoA carboxylase (ACC) [13] is elegantly discussed in the review by von Loeffelholz et al. [14]. Chronic caloric oversupply (e.g., hyperglycemia) may lead to the suppression of AMPK activity, promoting the activation of ACC and the deposition of lipids in the liver [14,15,16]. Several clinical trials are being conducted using metformin and other AMPK activators in combination with other anti-diabetic drugs to suppress de novo lipid production in NAFLD [17,18,19].

Both transcriptional and genetic alterations in AMPK pathways may impact tumor microenvironments in a tissue-dependent manner either positively or negatively [20]. This aspect of AMPK in breast cancer is reviewed in detail by Uprety and Abrahamse [21]. The authors discuss various pharmacological agents and strategies which activate AMPK signaling and examine their effects on cancer cells. The authors particularly discuss the potential use of vanadium compounds in anti-cancer therapy. Vanadium compounds potentially inhibit protein tyrosine phosphatase 1B (PTP1B) which is an endogenous inhibitor of AMPK. Thus, by relieving the suppression of AMPK by PTP1B, vanadium compounds indirectly activate AMPK. Therefore, vanadium compounds offer an alternative to direct-AMPK activators to treat cancer and diabetes as a combination therapy with other drugs.

Lastly, Bhutta et al. [22] reviewed the potential use of drugs that modulate AMPK activity as an anti-viral therapy. The authors discussed several studies conducted in cell culture or animal models showing beneficial or detrimental effects of AMPK activators on infection/proliferation of various viruses such as HBV, HCV, HMCV, HSV-1, KSHV, RSV, and SARS-CoV-2. The authors suggest that the use of activators or inhibitors of AMPK signaling may be beneficial depending on the type of virus. Several clinical trials are currently underway regarding the potential use of metformin as an add-on drug in HBV- and HCV-infected patients.
